# Doctors and International Peace

**Published:** 1911-03-04

**Authors:** 


### The Hospital

A Journal of

The Medical Sciences and Hospital Administration.

New Series. No. 213, Vol. VIII. [No. 1285, Vol. XLIX] SATURDAY, MARCH 4 1911.

### Doctors and International Peace.

During the last half-century the medical profession has slowly and gradually found out that it possesses great powers for good. And yet the old adage " Sub sole nil novi " remains good in this particular as in most others, for centuries ago the medical colleges in Egypt, where the professional lamp was first kindled, concerned themselves very actively with great public questions of the day. It is immaterial here to discuss the reasons for this recrudescence of interest in such subjects: we can merely be thankful that there is growing in the profession a desire to take a more active part in dealing with matters in which hitherto the lawyers, the statesmen, the priests, and the politicians have had a monopoly. Nor is it necessary to detail in what particular directions this increased interest has been manifested. We need only point to the share that the profession as a whole, and certain sections of it in particular, have taken in bringing about an amelioration of existing conditions of employment, of food production, of the education of the young, and of life generally. When the full history of the last century is written, the part played by medical men in sociological progress, particularly in the domain of State hygiene, will be gratefully acknowledged by the historian, and there is every reason to anticipate that the part the profession has yet to play in these matters during the coming decades will be immeasurably greater.

Of late years there has been a more determined, and let us add a more organised attempt to lessen the factors which tend to promote international unrest. When it is borne in mind that with very few exceptions all wars, and more especially modern wars, have been caused by social-economic conditions, it will readily be imagined that the influence of the medical profession may be of the greatest assistance in helping that movement. The profession, as such, has nothing to gain from international rivalry which is manifested in war. It has everything to gain from a rivalry which is shown by amicable competition. On the other hand, the medical man is in a particularly favourable position to discount the sentimental arguments which are advanced, on the one hand, for a scheme of general international peace, and on the other for a Chauvinistic programme which must inevitably lead to war. Once the social-economic problems have been cleared out of the way, the appeal to arms which is the last resort in grave questions of international right and wrong can be discussed in its true ethical aspects, but until that is done a vast amount of work remains—work in which the medical profession must do its share, since as a profession it is particularly suited to engage upon it. We, as medical men, know, better perhaps than our patients, the truth that underlies Tennyson's passionate utterance:—

> Peace in the vineyard, yes—but a company forges the wine!

We know, too, that the stagnation and misery bred from harsh and unfavourable conditions of life, from overcrowding and insanitation, are capable of being cured by other means than by engaging in a war, or, after the manner of savage tribes, by indulging in a witch hunt whereby the surplusage of population is weeded out. Gradually we are beginning to realise that the true measure of statesmanship lies in the manner in which these evils are treated, and that the drastic cutting of knots is much inferior to the patient, though necessarily slow, unravelling of tangles.

That being the case, medical men must feel particularly interested in the attempts which have been made from time to time to organise an International Society of Doctors who are pledged to do their utmost to promote peace. During the late Russo-Japanese war such an Association was formed at Paris, mainly through the tireless energy of Dr. J. Riviere, with whom were associated many prominent leaders of the profession in all civilised countries. This Committee, which is styled " The International Medical Association for the Suppression of War," has been steadily growing. The work that the Association has been doing is carried on quietly and unostentatiously by means of papers and meetings and an annual Congress. The next Congress will be held at Paris during September of this year, and the Committee appeals to all medical men who feel an interest in the matter to join the Association and to assist the work that is being done. The President of the Association is Dr. J. Rivière, 25 Rue des Mathurins, Opéra, Paris, who will gladly forward full particulars to those who desire to become members. No subscription is asked for or required, since the expenses of propaganda are defrayed by voluntary donations. All that is required is that medical men should signify their willingness to co-operate in the movement, which is entirely non-political, and which has for its object the attempt to promote solidarity and fraternity between the medical men of all nations in a manner that cannot effectively be done by means of triennial international medical congresses for purely scientific objects. We therefore commend the Association heartily to the attention of the profession in this country, in the hope that the next meeting in Paris will see the British Empire worthily represented among the speakers and participants of the Congress.

